# Association of erosive tooth wear and dental caries in Northern Finland Birth Cohort 1966 – an epidemiological cross-sectional study

**DOI:** 10.1186/s12903-016-0232-x

**Published:** 2016-07-04

**Authors:** Viivi Alaraudanjoki, Marja-Liisa Laitala, Leo Tjäderhane, Paula Pesonen, Adrian Lussi, Vuokko Anttonen

**Affiliations:** 1 Research Unit of Oral Health Sciences, University of Oulu, P.O. Box 5281, FI-90014 Oulu, Finland; 2 Medical Research Center Oulu, Oulu University Hospital and University of Oulu, Kajaanintie 54, FI-90029 Oulu, Finland; 3 Department of Oral and Maxillofacial Diseases, University of Helsinki and Helsinki University Hospital, Kasarmikatu 11-13, FI-00029 Helsinki, Finland; 4 Institute of Health Sciences, University of Oulu, P.O. Box 5281, FI-90014 Oulu, Finland; 5 Department of Preventive, Restorative and Pediatric Dentistry, University of Bern, Freiburgstrasse 7, CH-3010 Bern, Switzerland

**Keywords:** Erosion, Epidemiologic, Middle-aged, Dental caries

## Abstract

**Background:**

The main aim of this cross-sectional study was to investigate the prevalence and severity of erosive tooth wear and its association with dental caries and socio-demographic factors among middle-aged Finnish adults.

**Methods:**

Of the total Northern Finland Birth Cohort 1966 (*n* = 12,058), a convenience sample (*n* = 3181 adults) was invited for an oral health examination of which 1962 (61.7 %) participated, comprising the final study group. Clinical examinations were carried out by trained and calibrated dentists. Erosive tooth wear was assessed by sextants using the Basic Erosive Wear Examination Index (BEWE, 0–18) and dental caries at surface level using the ICDAS criteria (0–6). Socio-demographic data were obtained from a postal questionnaire. A logistic regression model was generated to test the association of the variables.

**Results:**

The prevalence of erosive tooth wear was 75 % and the mean of the BEWE sum score was 3.4 (SD 3.30). Almost half of the members needed non-invasive or invasive measures to prevent further progression of the condition. Of those with erosive lesions, 14.6 % suffered from severe erosive tooth wear. There was a strong positive relationship between the presence of severe erosive tooth wear (BEWE sum score ≥9) and male gender and restorative treatment need.

**Conclusions:**

Erosive tooth wear is a common finding in Finnish adult population; almost one in ten suffer from severe erosive tooth wear. Restorative treatment need seems to be associated with severe erosive tooth wear.

## Background

Tooth wear is conventionally categorized as dental erosion, attrition and abrasion. Dental erosion refers to tooth surface loss due to extended exposure to intrinsic or extrinsic acids. This process has been thought to be solely a tooth surface phenomenon, but it has been recently shown that erosive dissolution occurs also within the thin, partly demineralized and softened enamel layer [1] leaving the tooth surface vulnerable to mechanical forces. These forces remove the softened tooth surface, causing and exacerbating erosive tooth wear [2, 3]. Established risk indicators of erosive wear according to the recent studies include frequent or high consumption of acidic drinks and dietary products [2, 4–8] as well as acidic reflux [6, 7, 9] and hyposalivation [10]. However, it seems unlikely that one or two isolated factors are responsible for a multifactorial condition like erosive wear, instead, an interaction between chemical, mechanical and biological processes seems to be crucial [11]. Some of the risk indicators of erosive tooth wear are consistent with dental caries (i.e. frequent use of sweetened soft drinks and acidic candies) but interestingly, the role of some factors, such as socio-demographics [4, 12–14] and tooth brushing habits [6, 15] is still unclear and even suggested to be controversial.

Because of the numerous indices used for scoring erosive tooth wear, comparison between prevalence studies is difficult. Additionally, the number of epidemiological studies examining the condition in adults is limited and study set-ups differ remarkably, complicating comparisons and the ability to draw conclusions [16, 17]. The most recent epidemiologic studies in adult populations suggest a high prevalence of erosive tooth wear, ranging between 53 and 78 % [6, 18–20]. There is an indication that erosive tooth wear has become more prevalent in recent decades [21], which, however, is challenging to prove [11, 22].

Knowledge of erosive tooth wear in Finland is vague [6]. Therefore, the main purpose of this cross-sectional study was to investigate the prevalence and severity of erosive tooth wear among a Finnish adult population. Based on current research, the nature of erosive tooth wear and increasing proportion of aging individuals with own teeth [23], our hypothesis was that almost all in this age group have erosive changes of some degree. In addition, we investigated socio-demographic factors in association with erosive tooth wear and aimed to find out if the population in risk for dental caries also possesses a risk for erosive wear.

## Methods

### Study population

The study population was a part of the NFBC 1966, a birth cohort originally comprising all 12,058 children whose expected time of delivery was in the year 1966, and ultimately representing 96.3 % of all such births [24]. The entire NFBC 1966 has been evaluated regularly since birth by means of health questionnaires and clinical examinations. In connection with the 46-year follow-up survey (2012 – 2013), a subgroup (total of 3181 persons currently living in the city of Oulu or within 100 km of Oulu, including rural areas (convenience sample)) was asked to participate clinical oral examination. Of the original subgroup, a total of 1962 (61.7 %) participated and comprised the present study population.

### Dental examination

Between April 2012 and June 2013, oral examination was conducted using a standardized clinical dental examination protocol by 7 dentists. All examiners were trained and calibrated both before and during the course of the study. All examinations were carried out in a dental clinic with modern equipment and optimal lighting by a probe, oral mirror and WHO ball pointed gingival probe. The teeth were blow-dried using a three-in-one syringe before assessment. No professional cleaning was undertaken before the clinical examination, because the clinical examination also included periodontal measures. A dental nurse registered the findings in an individual electronic patient file (software designed for this study at the University of Oulu).

### Erosive tooth wear and dental caries – diagnostic criteria

Erosive tooth wear was measured using the BEWE index [25]. All tooth surfaces were examined and the highest score for each sextant was recorded. The scoring criteria were as follows: score 0 = no erosive tooth wear; score 1 = initial loss of surface texture; score 2 = distinct defect, hard tissue loss <50 % of the surface area; score 3 = hard tissue loss >50 % of the surface area. For scores 2 and 3, dentine is often involved. The sum scores of the sextant BEWE indexes were calculated (0–18). The examiners were advised not to include wedge-shaped defects or incisal edges in the examinations, if the role of abrasion or attrition was obvious. Reasons, when erosion could not be recorded were extensive restorations covering the entire tooth surface or prosthetic works. If there were less than two teeth in a sextant, the sextant was excluded from the analyses.

Caries lesions were detected using the International Caries Detection and Assessment System ICDAS [26]. ICDAS code 4 was set as the cut-off value for restorative treatment decision – i.e. lesions of that depth represents distinguished caries lesions. In borderline cases especially between ICDAS scores 3 and 4, the clinical examiners were advised to choose more severe option. In calculating DMFT values, ICDAS scores (D, ICDAS ≥ 4), presence of fillings (F) and extractions (M) were considered. The M value consisted of all missing teeth regardless of the cause of extraction since no data concerning previous dental treatments were available. No radiographs for specific caries detection were obtained.

Basic information was obtained with a survey designed for the entire cohort. Every cohort member received a paper copy of the questionnaire by mail, which was filled on paper. Socio-demographic data were collected from the questionnaires. For the present study, the specific variables obtained from the cohort data were gender (*n* = 1944), marital status (*n* = 1879) and education (*n* = 1869).

### Validation

The dentists performing the examinations were trained and calibrated by specialists and senior researchers on the field of cariology and periodontology. To ensure that all the examiners would have similar theoretical knowledge, lectures were given during the training sessions on the use of the ICDAS and BEWE classifications. Criteria for these were presented as a PowerPoint presentation on a screen using a PC and data projector. Following the lectures, and in order to practice their diagnostic skills, the dentists determined the classification of 26 extracted teeth with a variety of caries lesions using the ICDAS criteria. Revision of the criteria and calibration of the examiners were performed every third month. The criteria for the ICDAS and BEWE classifications were available to the examiners throughout the study in written and graphical form in the examination room. MLL, senior dentist and senior researcher, familiar with the study protocol and criteria, acted as a gold standard and assessed that the examiners followed the study protocol precisely throughout the entire field study.

To assess the intra-examiner agreement, the examiners re-examined (dental caries, BEWE) one quadrant of on average 10 patients approximately one month after the first examination. To assess the inter-examiner agreement, the gold standard (MLL) re-examined one quadrant of on average 12 patients from each examiner. Validation was carried out according to the guidelines given in the national Health 2000-study [23].

### Statistical issues

To describe and analyze the prevalence and severity of erosive tooth wear, the sum scores of BEWE index were calculated and interpreted as follows (adjusted from the original by combining the two highest risk groups): BEWE sum score 0–2 = no or only mild erosive tooth wear with no treatment need; BEWE sum score 3–8 = moderate erosive tooth wear, treatment needed; and BEWE sum score 9–18 = severe erosive tooth wear, treatment needed. The data were also analyzed by the highest BEWE score recorded per each individual (0–3). Wisdom teeth were excluded from the analyses.

The ICDAS codes were interpreted at tooth level as follows: score 0–3 = no restorative treatment need (D = 0); score 4–6 = need for restorative treatment (D = 1). To describe and analyze the prevalence and severity of caries among the study population at dentition level, restorative treatment need was first dichotomized as follows: no restorative treatment need (D = 0) and restorative treatment needed (D > 0). For further analyses, restorative treatment need was categorized as follows: D = 0 (no restorative treatment need), D = 1–3 (moderate restorative treatment need) and D ≥ 4 (high restorative treatment need). Existence of caries lesions was determined at sextant level to be able to compare dental caries and BEWE indices on sextant level. If any tooth in a sextant needed restorative treatment, the score for the sextant was determined to be 1. To analyze the association between past and present caries experience and erosive wear, DMFT indices were calculated and categorized. Based on the mean DMFT value among the cohort, DMFT ≤ 14 was considered as low and DMFT >15 as high caries experience. Association between past and present caries experience and erosive wear was analyzed by comparing BEWE sum scores and categorized (low and high) DMFT values.

Marital status was dichotomized into persons married, cohabiting and living in a registered relationship in one group, and the rest (unmarried, divorced, widowed) in the other. Education was categorized into basic (9 years of comprehensive school) and high school (total 12 years, matriculation examination) education.

Descriptive statistics such as frequencies, distributions, means and standard deviations were used. Cross-tabulation and chi-square tests were used for analyzing the distribution of different variables according to gender. Because of the skewness of the distribution, Mann-Whitney’s *U*-test was used to compare the number of decayed tooth and BEWE sum scores between genders. An independent sample *t*-test was used to compare the mean DMFT values between genders. The association between severe erosive tooth wear and different variables was analyzed using logistic regression models from which OR values and 95 % confidence intervals (CI) were calculated. All variables (gender, education, marital status, restorative treatment need, DMFT) were initially included in bivariate analyses (chi-square test and unadjusted logistic regression model), and the variables that were statistically significantly associated with erosive tooth wear were included in the adjusted logistic regression model. A *P*-value <0.05 was considered statistically significant. All statistical analyses were performed using SPSS (version 22.0, SPSS, Inc., Chicago, IL, USA).

## Results

Females dominated slightly the study population. Females also were distinctly more educated than males, whereas males had higher mean BEWE sum score and more restorative treatment need and past caries experience (Table 1).

**Table 1:** Background characteristics of the participants and descriptive statistics of outcome variables stratified by gender

Variables n (%)		Male n (%)	Female n (%)	Total n (%)	*p*-value
Education	Basic	554 (64.3)	438 (43.5)	992 (53.1)	<0.001
	High	307 (35.7)	570 (56.5)	877 (46.9)	
Marital status	Married/cohabiting	694 (80.2)	779 (76.8)	1473 (78.4)	0.07
	Single	171 (19.8)	235 (23.2)	406 (21.6)	
Restorative treatment need	No	482 (53.4)	688 (66.1)	1170 (60.2)	<0.001
	Yes	421 (46.6)	353 (33.9)	774 (39.8)	
Number of decayed tooth, mean (SD)		1.2 (2.0)	0.8 (1.7)	1.0 (1.9)	<0.001
DMFT, mean (SD)		15.3 (5.4)	14.6 (4.9)	14.9 (5.1)	0.006
BEWE sum score, mean (SD)		3.8 (3.5)	3.1 (3.1)	3.5 (3.3)	<0.001
Total		903 (46.5)	1041 (53.5)	1944 (100)	

The difference between genders have been calculated using chi-square test and it was statistically significant (*p* < 0.05) considering all variables except between marital status

Three in four had erosive tooth wear *per se*. About half had erosive wear needing preventive measures or operative care (Table 2). According to the highest BEWE score for each individual, the highest value was 1 for 54.7 %, 2 for 18.2 % and 3 for 2.1 %. Males dominated both as for the prevalence and the severity of erosive tooth wear (Table 2). Distinct erosive defects (BEWE class 2 or 3) were most prevalent in maxillary and mandibular anterior sextants (15.0 %). In other sextants, the prevalence of significant tissue loss varied from 5.8 to 6.3 %. There was considerably more distinct erosive wear in anterior sextants in the male (19.8 %) than in the female population (10.8 %) (*p* < 0.001).

**Table 2:** The prevalence of erosive tooth wear stratified by gender (age range 44–46 years)

BEWE sum score	Male n (%)	Female n (%)	Total n (%)	*p*-value
0	205 (22.7)	282 (27.1)	487 (25.1)	0.026
1–2	222 (24.6)	302 (29.0)	524 (27.0)	0.028
3–8	390 (43.2)	407 (39.1)	797 (41.0)	0.067
9–18	86 (9.5)	50 (4.8)	136 (7.0)	<0.001
Total	903 (100)	1041 (100)	1944 (100)	

The differences between genders concerning BEWE sum scores have been calculated using chi-square test and were statistically significant (*p* < 0.05), except for BEWE sum scores 3–8

Among the study population, moderate to severe erosive tooth wear was most prevalent in males with only basic education (53.6 %) and least prevalent among women who had completed a matriculation examination (40.2 %) (*p* < 0.001). Married or cohabiting women had significantly less (41.3 %) moderate or severe erosive tooth wear than single women (52.3 %) (*p* < 0.003). The marital status of men showed no association with erosive tooth wear.

The mean DMFT score in the entire study population was 14.9 (SD 5.15) (Table 1). The proportion of adults in need of restorative treatment was 39.8 %, and the mean value for D was 1.0 (SD 1.84). The association between restorative treatment need and moderate and severe erosive tooth wear was statistically significant, whereas the association between erosive wear and DMFT was statistically significant only concerning sum scores ≥3. In the group of individuals with high restorative treatment, 12.2 % had severe erosive wear, compared to 4.9 % of those with no restorative treatment need at all (*p* < 0.001) (Table 3). At the sextant level, there was a statistically significant positive association with severe erosive tooth wear (BEWE index 2–3) and restorative treatment need in sextants 1 and 5 (*p* < 0.03).

**Table 3:** The association between severe erosive wear and independent variables. Unadjusted and adjusted logistic regression models

Variable		Severe erosive wear (BEWE sum score ≥9)					
		No n (%)	Yes n (%)	*p*-value*	Unadjusted OR (95 % CI)	Adjusted OR^a^(95 % CI)	Adjusted OR^b^(95 % CI)
Gender	Female	991 (95.2)	50 (4.8)	<0.001	1	1	1
	Male	817 (90.5)	86 (9.5)		2.1 (1.46–2.99)	1.8 (1.26–2.68)	1.8 (1.25–2.65)
Education	High	827 (94.3)	50 (5.7)	0.037	1	1	1
	Basic	911 (91.8)	81 (8.2)		1.5 (1.02–2.12)	1.1 (0.77–1.65)	1.1 (0.75–1.62)
Marital status	Married/cohabiting	1372 (93.1)	101 (6.9)	0.709	1	Not included	Not included
	Single	376 (92.6)	30 (7.4)		1.1 (0.71–1.66)		
Restorative treatment need	No	1113 (95.1)	57 (4.9)	<0.001	1	1	Not included
	Yes	695 (89.8)	79 (10.2)		2.2 (1.56–3.16)	2.1 (1.47–3.08)	
Restorative treatment need	No	1113 (95.1)	57 (4.9)	<0.001	1	Not included	1
	Moderate^c^	551 (90.3)	59 (9.7)		2.1 (1.43–3.05)		2.0 (1.34–2.94)
	High^d^	144 (87.8)	20 (12.2)		2.7 (1.58–4.64)		2.7 (1.56–4.75)
DMFT	≤14	885 (93.6)	61 (6.4)	0.357	1	Not included	Not included
	>14	923 (92.5)	75 (7.5)		1.2 (0.83–1.67)		

*p-values, chi-square test

^a^logistic regression model computed using dichotomized restorative treatment need

^b^logistic regression model computed using trichotomized restorative treatment need

^c^D = 1–3

^d^D ≥ 4

There was a strong positive relationship between severe erosive tooth wear (BEWE sum score ≥9) and restorative treatment need and gender (Table 3). The adjusted OR for severe erosive tooth wear was 1.8 for males (95 % CI 1.26 to 2.68), 2.1 for those with restorative treatment need (95 % CI 1.47 to 3.08) and 2.7 for those with high restorative treatment need (95 % CI 1.56 to 4.75). The level of education was statistically significant in an unadjusted regression model, but no longer in the adjusted model.

To describe the intra- and inter-examiner agreement considering individuals affected with erosive tooth wear of any degree, Cohen’s kappa values were calculated based on the re-examined sextants. The mean kappa value for the intra-examiner agreement was 0.46, and for the inter-examiner agreement between the gold standard and the examiners 0.30. When considering agreement on distinct erosive wear (BEWE class 2 or 3), the mean intra-examiner agreement was 0.98 and the inter-examiner agreement 0.81. Considering restorative treatment need, the mean kappa value for the intra-examiner agreement was 0.64 and for the inter-examiner agreement 0.61.

## Discussion

Among the NFBC 1966 participants, the overall prevalence of erosive tooth wear was high. Almost half of the now middle-aged cohort members were found to be in need of at least preventative measures against further progression of the condition. Of the individuals with erosive lesions, 14.6 % suffered from severe erosive tooth wear. The risk for severe erosive wear was two-fold for males and those with restorative treatment need.

The most recent studies using the BEWE index support the present prevalence figures [4, 6, 20]. In a large scale multicenter study (including Finland), the overall prevalence of at least one erosive lesion in a sample of European young adults was 57.1 % and of the Finnish participants 17.7 % had a maximum single BEWE score of 2 or more [6]. The subjects were younger than here, which may explain the slightly higher prevalence observed in the present study. Furthermore, the predominance of erosive tooth wear in the upper anterior sextant has also been recently reported [20]. Despite the differences in the scoring systems in other recent studies [27, 28] the prevalence values reported are similar to the present study.

According to this study, those with high restorative treatment need were at almost three-fold risk for severe erosive wear. In addition, past caries experience was positively associated with present erosive wear. Both dental caries and erosive tooth wear have common etiological factors, such as high or constant intake of sweetened soft drinks, low saliva secretion and unhealthy dietary habits, which may explain the association. Furthermore, individuals with erosion have suggested having salivary characteristics similar to those of caries-active individuals [29]. A clinical implication might be, that current restorative treatment need status may act as a risk indicator for erosive wear, and vice versa. However, findings in the literature on the issue are equivocal, with many studies reporting statistically significant association between erosive tooth wear and dental caries [27, 30, 31] while others do not [12, 32].

With regard to gender differences, the prevalence of erosive tooth wear was more frequent in males than in females. This is in agreement with many previous reports [7, 13, 15, 28, 32]. Yet, there are still many studies that have reported no gender differences in the prevalence of erosive tooth wear [6, 20]. As a biological explanation for males being more prone to erosive tooth wear, it has been suggested that there are differences between genders in the consumption of carbonated drinks and in the strength of biting forces [33]. Indeed, tooth grinding may be significantly associated with the incidence of erosive tooth wear and may play a major role in erosive lesions – bigger than has been suspected [2]. This needs further investigations.

In the present study, socio-demographic variables were associated with erosive wear among females, and lower education was associated with elevated risk for severe erosive wear in unadjusted regression model. However, education was not found to be statistically significant variable for severe erosive tooth in the adjusted logistic regression model. With regard to other studies, many have reported no significant association between the condition and sociodemographic factors [6, 20, 30]. One study reported erosive tooth wear being more common among individuals rated with a higher socioeconomic status [14]. Both healthy as well as unhealthy diets may contain acidic foods, thus adults from different socioeconomic backgrounds could possess a similar risk for erosive tooth wear. The overall lack of statistical significance with respect to socio-demographic factors may also be explained by the cross-sectional nature of many studies, including the present one. Given that erosive tooth wear usually develops over time, lifestyle factors present for several years may have been responsible for the current erosive tooth wear. Long-term effects of dietary habits in association with erosive tooth wear could be a topic for a future study.

The strength of this study was a big study population, not limited to any subgroup. Thanks to the large enough sample size, our statistical power is good. Also participation rate was relatively high (62 %). Concerning caries experience, our results are in line with Health 2000-survey [23], thus this sample can be considered to represent well Finnish adults. However, the presence of response bias must always be kept in mind in cohort studies. Another limitation is the fact that differential diagnosis of erosive wear is difficult, as it usually co-exists simultaneously with other types of tooth wear, especially in the ageing dentitions, such as in the present study. Diagnosis of early signs and symptoms of erosive tooth wear is challenging even in dentitions without other forms of wear. Even though we made attempts to distinguish between erosion, attrition and abrasion, the examiner’s individual opinion on the origin of wear may have produced bias in the study. In addition, with respect to caries diagnostics, the ICDAS protocol states that the teeth should be cleaned with at least a toothbrush and floss before conducting an examination [26]. In our case, no professional cleaning was undertaken; however it is a common habit to brush teeth before seeing a dentist in Finland. It is also well known that radiography may give a substantial contribution to the diagnosis of interdental and occlusal caries. PTG radiographs were taken of all individuals, but they are not reliable in caries diagnostics.

The BEWE index itself and the use of the BEWE cumulative score have been validated and shown to be acceptable for recording tooth wear and scoring severity and in prevalence studies [34, 35].

However, in the study of Olley et al. (2014) [35], the BEWE index was used as a tool for diagnosing tooth wear overall not distinguishing between erosion and mechanical wear. In our study, the examiners had difficulties in differentiating between intact enamel and initial loss of surface texture. Same tendency can be seen in other studies, especially when having multiple examiners [34, 36]. A recent study of the validity and reliability of the BEWE index showed only a moderate inter- and intra-examiner agreement [37] suggesting that the BEWE scores should be interpreted with some caution. However, the authors concluded that BEWE is an appropriate and reliable tool. Despite the given sufficient accuracy of the BEWE scoring system, the problems in diagnosing mild erosive tooth wear in aged dentitions must be borne in mind. Using the BEWE sum score and the cut-off point ≥9 for analyses is likely to increase the reliability of the study. In addition, the cut-off values have been suggested to be in need of re-evaluation [38]. A study evaluating the reliability and accuracy of the BEWE scoring system in adult population for erosive tooth wear, would be most valuable.

With respect to the BEWE sum scores, the cut-off values were based on the experience and previous studies of one of the authors (AL), and this topic is under on-going consideration and evaluation [25]. Vered et al. (2014) [20] suggested that the cut-off sum score for high risk could be 7, for medium risk 4–6 and for low risk 1–3. Originally, the cut-off value for high risk was 14, for medium risk 8–13, for low risk 3–6 and for no risk 0–2 [25]. Another option is to use only the highest BEWE score per subject, but it may not be as comprehensive as the BEWE sum score. Whether using the BEWE sum score (0–18) or the highest BEWE score per subject (0–3), the outcomes in the present study remained substantially the same. Therefore, according to the present results, either system can be used.

## Conclusion

In conclusion, erosive tooth wear appears to be common condition in a middle-aged Finnish population. Male gender appeared to increase the risk for erosive tooth wear, whereas other socio-demographic factors do not seem to be associated with it in this population. Restorative treatment need seems to be associated with erosive wear.

## Abbreviations

BEWE, basic erosive wear examination; CI, confidence interval; DMFT, decayed, missing, filled teeth; ICDAS, International Caries Detection and Assessment System; NFBC, Northern Finland Birth Cohort; OR, odds ratio; SD, standard deviation; WHO, World Health Organization
